# Performance Degradation and Service Life Prediction of Magnesium Oxychloride Cement Recycled Concrete in Western Saline Soil Environment

**DOI:** 10.3390/ma19122672

**Published:** 2026-06-22

**Authors:** Shijie Chai, Nan Wang, Yuze Tian, Wei Gong, Peng Yin

**Affiliations:** 1 School of Civil Engineering, University of Science and Technology Liaoning, Anshan 114051, China; 2 School of Civil Engineering and Architecture, Jiangsu University of Science and Technology, Zhenjiang 212100, China; 3 Faculty of Infrastructure Engineering, Dalian University of Technology, Dalian 116023, China

**Keywords:** magnesium oxychloride cement recycled concrete, compressive strength, size effect, environmental error, time-varying degradation model, service life

## Abstract

**Highlights:**

- The durability of MOCRC in a western saline soil environment is investigated.
- A multiparameter time-varying degradation model for MOCRC is established.
- A service life prediction of MOCRC is proposed.

**Abstract:**

Western saline soil areas contain a large amount of chloride and sulfate ions, leading to a reduction in the service life of Portland concrete in this environment. Magnesium oxychloride cement recycled concrete (MOCRC) is mainly prepared with light-burnt magnesia and magnesium chloride, which is more suitable for application in a western saline soil environment than Portland concrete. In this paper, ultrasonic non-destructive testing technology was used to investigate the effects of multiple factors on the deterioration process and service life of MOCRC in a western saline soil environment. The results showed a clear functional relationship between the relative dynamic elastic modulus and the compressive strength or size of MOCRC. On this basis, a multiparameter time-varying degradation model for MOCRC considering the compressive strength, size effect, and environmental error is established. Moreover, a service life prediction model for MOCRC based on the relative dynamic elastic modulus is proposed, using reliability theory and the first-order second-moment method. This study provides a foundational method for the durability examination and service life prediction of MOCRC.

## 1. Introduction

With the development of salt lake areas, the demand for infrastructure construction continues to increase. Magnesium oxychloride cement (MOC) is a low-carbon cementitious material produced by the reaction between light-burnt magnesia and magnesium chloride solution [1,2,3], as shown in Equations (1) and (2). Compared with Portland cement concrete, magnesium oxychloride cement concrete (MOCC) produced with MOC has high mechanical strength, high- and low-temperature resistance, and salt corrosion resistance [4,5]. MOCC is not only suitable for cold regions, saline soil areas, and salt lake areas, but also contributes to solving the problem of “magnesium damage” in western saline soil environments.

5MgO + MgCl_2_ + 13H_2_O → 5Mg(OH)_2_⋅MgCl_2_⋅8H_2_O (1)

3MgO + MgCl_2_ + 11H_2_O → 3Mg(OH)_2_⋅MgCl_2_⋅8H_2_O (2)

The application of MOCC in western saline soil environments faces problems such as high raw material costs and large consumption of natural sand. Recycled aggregate is a granular material made from concrete, mortar, stones, bricks and tiles from decoration waste through sorting, crushing and other processes [6,7,8]. The replacement of natural sand and gravel with recycled aggregate to prepare magnesium oxychloride cement recycled concrete (MOCRC) can effectively reduce the raw material costs. However, recycled aggregate exhibits high water absorption, high porosity, and low mechanical strength compared to natural aggregate. Studies [9,10] show that the use of recycled aggregates has a negative impact on the compressive strength of concrete, thereby affecting its durability. Notably, previous studies on the durability of MOC have focused mainly on water resistance improvement without involving recycled aggregate [11,12]; the combination of MOC and recycled aggregate may lead to effects due to changes in hydration environment and phase stability. Size effect and environmental error (differences between the simulated and actual environments) also have an influence on the durability of concrete [13,14,15]. Therefore, a multiparameter time-varying degradation model considering the compressive strength, size effect, and environmental error can be established for MOCRC.

Based on the failure principles of concrete structures, the service life of Portland cement concrete is usually predicted using the diffusion behavior of chloride ions [16,17]. The chloride ion content of MOCC ranges from 1.5% to 6.0% [18], far exceeding the critical value for rebar corrosion (0.05–0.5%) [19,20], which leads to the premature corrosion of reinforced steel. To address this issue, stainless steel [21] or basalt fiber [22] are commonly used in MOCC. The poor water resistance of MOC is also unfavorable to the durability of MOCC [11,12]. However, existing service life prediction models for MOC—whether based on chloride diffusion [18,19] or gray system prediction [22]—cannot be directly applied to MOCRC, because they do not account for environmental error, size effects, or the high internal chloride content of MOC, nor do they incorporate reliability theory. Therefore, it is necessary to investigate the degradation process of MOCRC to evaluate its durability and service life.

To assess the durability of MOCRC in western saline soil environments, ultrasonic non-destructive testing technology is used to examine the effects of multiple factors on the service process of MOCRC in this paper. A multiparameter time-varying degradation model for MOCRC is established based on the exposure and simulation experiments, combined with the compressive strength, size effect, and environmental error. Moreover, a service life prediction model for MOCRC using the relative dynamic elastic modulus (RDEM) is proposed based on reliability theory and the first-order second-moment method.

## 2. Materials and Methods

### 2.1. Materials

The main materials used in this paper include light-burnt magnesia (LBM, from Haicheng Fengchi Refractory Materials Co., Ltd., Haicheng, China) with an activity of 60%; industrial magnesium chloride (MC, from Tianjin Shuangrong Chemical Trade Co., Ltd., Tianjin, China) with a content of 96.8% MgCl_2_·6H_2_O; fly ash (FA, from Haicheng Junjing Trading Co., Ltd., Haicheng, China) with a total oxide content (SiO_2_ + Al_2_O_3_) of 83%; sand with a fineness modulus of 2.7; coarse aggregate (CA) and recycled coarse aggregate (RCA) with particle sizes ranging from 5 mm to 20 mm (derived from waste Portland cement concrete of grade C30); industrial phosphoric acid (PA, from Tianjin Ruijinte Chemical Co., Ltd., Tianjin, China) with a content of more than 85% H_3_PO_4_; naphthalene superplasticizer (NS, from Hubei Qiangda Chemical Co., Ltd., Jingzhou, China) with a water-reducing ratio of 21% and 0.5% Na_2_SO_4_; and water. The chemical composition and performance indicators of raw materials are listed in Table 1 and Table 2.

### 2.2. Specimen Preparation

The mix proportions and 28-day compressive strength of MOCRC specimens are shown in Table 3. Prior to mixing, RCA was pretreated to a saturated surface-dry condition by soaking in water for 24 h. The preparation process of MOCRC specimens is as follows: (1) weighing the materials (LBM, NS, MC, FA, PA, sand, water, CA, and RCA); (2) preparing the liquid mixture by mixing MC, NS, and PA with water for one minute; (3) preparing the solid mixture by mixing LBM, sand, FA, CA, and RCA for one minute; (4) mixing the mixture from steps (2) and (3) for three minutes to obtain the fresh concrete, which was poured into molds of 100 mm × 100 mm × 100 mm, 100 mm × 100 mm × 300 mm, and 100 mm × 100 mm × 400 mm; (5) demolding after 24 h, and curing for 28 days indoors at a temperature fluctuating between 14 °C and 22 °C and a relative humidity fluctuating between 59% and 69%.

For convenience, C35-100, C30-100, C25-100, and C20-100 represent 100 mm × 100 mm × 100 mm cube specimens with strength grades of C35, C30, C25, and C20, respectively. For each mix ratio, nine specimens were prepared and divided into three groups of three: the first group was tested for 28-day compressive strength; the second group was tested for XRD patterns at 1 year; the third group was tested for RDEM at different service ages and subsequently for XRD patterns at 3 years. C35-300 and C35-400 indicate the dimensions of 100 mm × 100 mm × 300 mm and 100 mm × 100 mm × 400 mm prism specimens with a strength grade of C35, with three specimens prepared for each size, tested only for RDEM.

### 2.3. Experimental Scheme

In this paper, MOCRC specimens were fully embedded in a simulated western saline soil environment to replicate actual field exposure. According to reference [23], the chemical composition of soil in a western saline soil environment is shown in Table 4.

### 2.4. Data Collection

The durability test of MOCRC specimens in a simulated western saline soil environment was carried out. The data collection cycle was 0.25 years-long and the total age was 3.0 years. The ultrasonic testing analyzer (Beijing Koncrete Testing Technology Co., Ltd., Beijing, China) was used to collect data on the ultrasonic velocity of MOCRC through the opposite measurement method [24,25]. RDEM is a mechanical performance parameter that represents the concrete damage. RDEM can be obtained as shown in Equation (3) [24].

(3) $$E_{r}=\frac{V_{t}^{2}}{V_{0}^{2}}$$

where *E*_r_ is the RDEM at the service age of t; *V*_t_ and *V*_0_ are the ultrasound velocity after and before the durability test, respectively; and *t* is the experimental age of durability test.

XRD measurement of MOCRC specimens was conducted on a PANalytical X’Pert Powder diffractometer (Malvern Panalytical B.V., Almelo, The Netherlands) with Cu Kα radiation over a 2θ range of 5–70°.

## 3. Results

### 3.1. Deterioration of MOCRC Based on the RDEM

**Figure 1:**
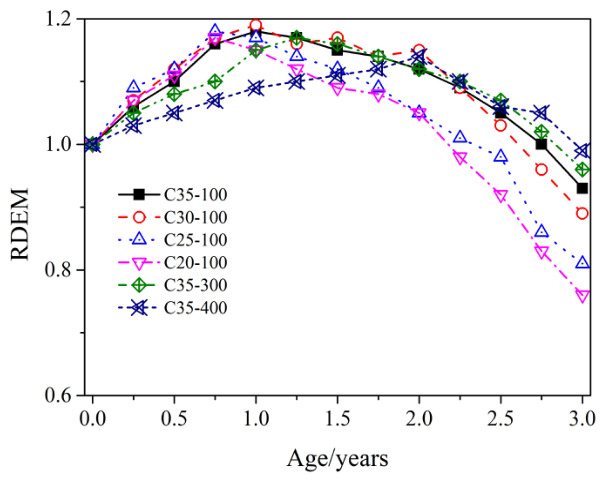
Time-varying characteristics of the RDEM of MOCRC specimens.

**Figure 2:**
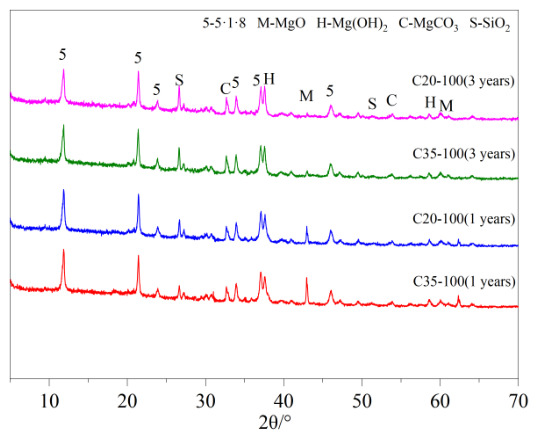
XRD patterns of MOCRC specimens cured for different ages.

The size effect is generated by the changes in length of MOCRC specimens in this paper. For easy understanding, the size is recorded as length. The relationship between the RDEM and service ages of MOCRC specimens with different compressive strengths and lengths in a simulated western saline soil environment is shown in Figure 1. It can be seen from Figure 1 that the RDEM of MOCRC specimens first increases and then decreases with the increase in service ages. This initial increase is attributed to the continued formation of the 518 phase, and pore refinement from ongoing hydration before long-term phase transformation dominates. The strength of MOCRC specimens is closely related to the MOC. As seen in Figure 2, the phase composition of MOCRC specimens at 1 year is mainly composed of 5Mg(OH)_2_⋅MgCl_2_⋅8H_2_O (518 phase), Mg(OH)_2_, unreacted MgO, and minor amounts of MgCO_3_ and SiO_2_. After serving for 3 years, the peak intensity of the 518 phase and MgO decreases, while the peak intensity of Mg(OH)_2_ increases. On the one hand, the 518 phase is unstable in humid environments, and transformed into 3Mg(OH)_2_·MgCl_2_·8H_2_O (318 phase), as shown in Equation (4). The 318 phase will be further decomposed to Mg(OH)_2_, as shown in Equation (5). Mg(OH)_2_ with poor crystallinity has low strength. On the other hand, the transformation of unreacted MgO into Mg(OH)_2_ generates the stress, due to the different densities of MgO and Mg(OH)_2_. These are the main reasons for the decrease in the RDEM and the compactness of MOCRC specimens.

5Mg(OH)_2_⋅MgCl_2_⋅8H_2_O → 3Mg(OH)_2_⋅MgCl_2_⋅8H_2_O + 2Mg(OH)_2_ (4)

3Mg(OH)_2_⋅MgCl_2_⋅8H_2_O → 3Mg(OH)_2_ + MgCl_2_ + 8H_2_O (5)

Figure 1 also shows that the effect of compressive strength on the RDEM of MOCRC specimens becomes more significant with the increase in service ages. At a service age of 3.0 years, the RDEM of C30-100, C25-100, and C20-100 decreases by 4.3%, 12.9%, and 18.3% compared with C35-100, respectively. Studies [26] show that the long-term test results can better reflect the durability of concrete. Moreover, the RDEM of MOCRC specimens increases with the increase in length. A possible explanation is that longer specimens may reduce the rate of water and ion ingress from the exposed surfaces, thereby slowing phase decomposition, a size effect captured by RDEM as an average damage indicator; however, this hypothesis requires further investigation using direct moisture transport measurements.

### 3.2. Multiparameter Time-Varying Deterioration Model for MOCRC

#### 3.2.1. Model

**Figure 3:**
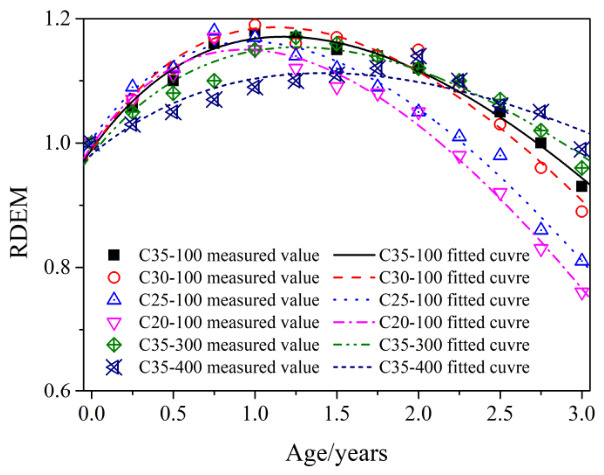
Time-varying degradation fitting curves of MOCRC specimens.

Based on the data in Figure 1, Equation (6) is used to perform the nonlinear fitting on the time-varying deterioration of RDEM in MOCRC specimens. The fitting results of curves are shown in Figure 3.

(6) $$E_{r}=a\times \ln\left(t+b\right)+c\times t$$

where *a*, *b*, and *c* are the fitting parameters.

According to Table 5 and Figure 3, the determination coefficients (Adj. R^2^) of time-varying degradation fitting curves for C35-100, C30-100, C25-100, C20-100, C35-300, and C35-400 are 0.9786, 0.9664, 0.9779, 0.9847, 0.9360, and 0.8921, respectively. The results show that the time-varying law of RDEM in MOCRC specimens conforms to Equation (6). In addition, the compressive strength and length of MOCRC specimens are related to the parameters of fitting curves: as the compressive strength or length increase, “a” decreases, while “b” and “c” increase. Considering the influence of the compressive strength and length of MOCRC specimens, as well as the differences between simulated and real environments, the multiparameter time-varying model for the degradation of MOCRC specimens is provided in the following equation.

(7) $$E_{r}=K_{f1}K_{L1}K_{e1}\ln\left(t+K_{f2}+K_{L2}+K_{e2}\right)+K_{f3}K_{L3}K_{e3}t$$

where *K_f_*_1_, *K_f_*_2_, and *K_f_*_3_ are the partial factors of compressive strength on RDEM. *K_L_*_1_, *K_L_*_2_, and *K_L_*_3_ are the partial factors of length on RDEM. *K_e_*_1_, *K_e_*_2_, and *K_e_*_3_ are the partial factors of environment on RDEM.

#### 3.2.2. Parameters

(1) Compressive strength

Equation (7) can be expressed into Equation (8), only considering the effect of compressive strength on the RDEM of MOCRC specimens.

(8) $$E_{r}=K_{f1}\ln\left(t+K_{f2}\right)+K_{f3}t$$

**Figure 4:**
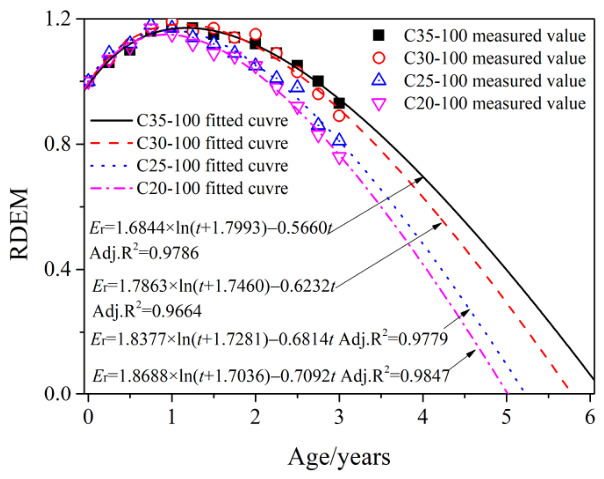
Time-varying degradation of MOCRC specimens with different compressive strengths.

**Figure 5:**
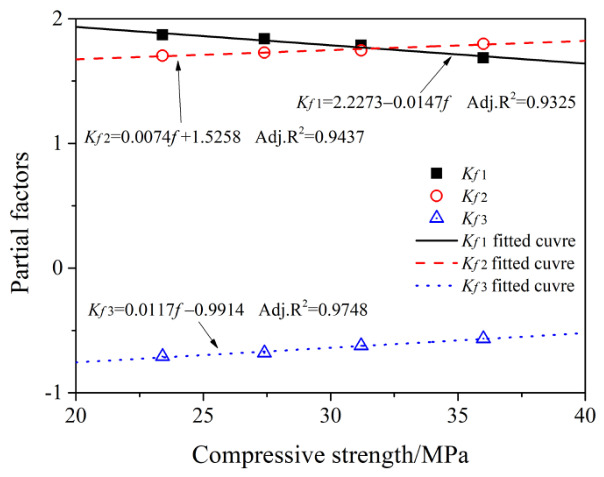
Relationship between the compressive strength and partial factors.

Based on the data of C35-100, C30-100, C25-100 and C20-100 in Figure 1 and Equation (8), the time-varying degradation of MOCRC specimens in a simulated western saline soil environment with different compressive strengths is shown in Figure 4. It can be seen in Figure 4 that the Adj. R^2^ of time-varying degradation fitting curves for C35-100, C30-100, C25-100, and C20-100 are 0.9786, 0.9664, 0.9779, and 0.9847, respectively. This confirms that the time-varying law of RDEM in MOCRC specimens with different compressive strengths conforms to Equation (7). Figure 5 also shows a clear linear functional relationship between the compressive strength and partial factors.

(2) Length

Equation (7) can be substituted into Equation (9), considering the effect of compressive strength and length on the RDEM of MOCRC specimens in a simulated western saline soil environment.

(9) $$\begin{array}{l} E_{r}=\left(2.2273-0.0147f\right)K_{L1}\ln\left(t+0.0074f+1.5258+K_{L2}\right)\\ +\left(0.0117f-0.9914\right)K_{L3}t \end{array}$$

**Figure 6:**
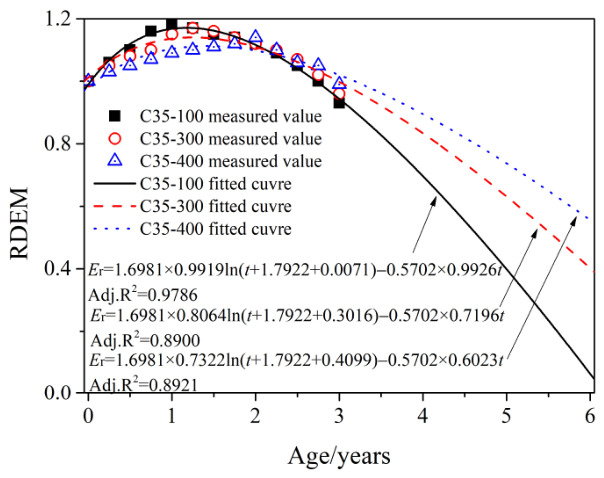
Time-varying degradation fitting curves of MOCRC specimens with different lengths.

**Figure 7:**
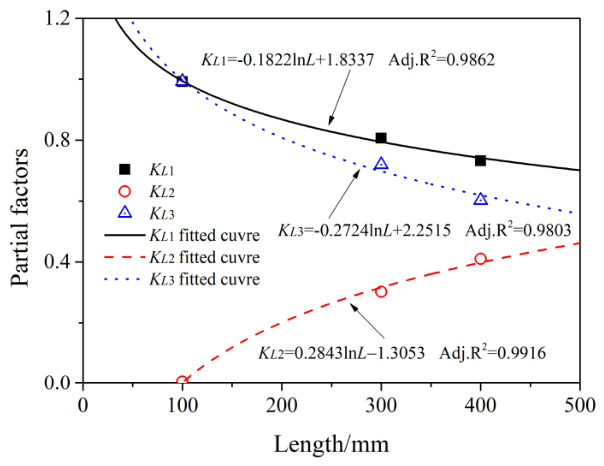
Relationship between the length and partial factors.

Based on the data of C35-100, C35-300 and C35-400 in Figure 1 and Equation (9), the time-varying degradation of MOCRC is shown in Figure 6. It can be seen from Figure 6 that Adj. R^2^ values of time-varying degradation fitting curves for C35-100, C35-300, and C35-400 are 0.9786, 0.8900, and 0.8921, respectively. This means that the time-varying law of the RDEM of MOCRC specimens with different lengths conforms to Equation (9). Figure 7 also shows a clear logarithmic functional relationship between the length and partial factors.

(3) Environmental error

Equation (7) can be substituted into Equation (10), considering the effect of compressive strength, length and environment on the RDEM of MOCRC specimens.

(10) $$\begin{array}{l} E_{r}=\left(2.2273-0.0147f\right)\left(-0.182\ln L+1.8337\right)K_{e1}\ln\left(t+0.0074f+0.2843\ln L+K_{e2}+0.2205\right)\\ +\left(0.0117f-0.9914\right)\left(2.2515-0.2724\ln L\right)K_{e3}t \end{array}$$

**Figure 8:**
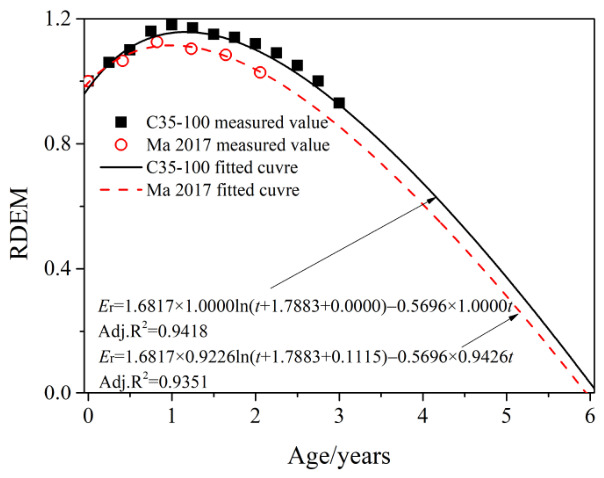
Time-varying degradation fitting curves of C35-100 in actual and simulated environments [27].

To compare the durability of MOCRC in the actual environment [27] and a simulated environment, the time-varying degradation fitting curves of C35-100 are shown in Figure 8. It can be seen from Figure 8 that the Adj. R^2^ of time-varying degradation fitting curves for C35-100 in the reference and in this paper are 0.9351 and 0.9418, respectively. The results show that the time-varying law of RDEM in MOCRC specimens conforms to Equation (10).

In summary, the multiparameter time-varying model for the degradation of MOCRC specimens is shown in the following equation:

(11) $$\begin{array}{l} E_{r}=0.9226\left(2.2273-0.0147f\right)\left(-0.182\ln L+1.8337\right)\ln\left(t+0.0074f+0.2843\ln L+0.332\right)\\ +0.9426\left(0.0117f-0.9914\right)\left(2.2515-0.2724\ln L\right)t \end{array}$$

### 3.3. Service Life Prediction of MOCRC

#### 3.3.1. Reliability Theory

The reliability theory is widely used in the durability of concrete structures. In this paper, the performance function (*Z*) based on the RDEM of MOCRC specimens is shown in Equation (12), where the critical value (0.60) [28] of RDEM is taken as the resistance (*E_cr_*) and the RDEM is taken as the effect (*E_r_*).

(12) $$Z=E_{r}-E_{\mathrm{cr}}$$

According to the basic principle of probability theory, the performance function conforms to a normal distribution if the performance function is a linear function of basic variable that follows a normal distribution. Therefore, when *E*_cr_ and *E*_r_ obey the normal distribution function, the mean (*μ_Z_*) and standard deviation (*σ_Z_*) of *Z* are shown in Equations (13) and (14), respectively.

(13) $$\mu_{Z}=\mu_{E_{r}}-\mu_{E_{\mathrm{cr}}}$$

(14) $$\sigma_{Z}=\sqrt{\sigma_{E_{r}}^{2}+\sigma_{E_{\mathrm{cr}}}^{2}}$$

The reliability index (*β*) and failure probability (*P*) of the service life prediction function for MOCRC specimens are shown in Equations (15) and (16), respectively.

(15) $$\beta =\frac{\mu_{Z}}{\sigma_{Z}}=\frac{\mu_{E_{r}}-\mu_{E_{\mathrm{cr}}}}{\sqrt{\sigma_{E_{r}}^{2}+\sigma_{E_{\mathrm{cr}}}^{2}}}$$

(16) $$P=\Phi \left(-\beta \right)=\Phi \left(-\frac{\mu_{E_{r}}-\mu_{E_{\mathrm{cr}}}}{\sqrt{\sigma_{E_{r}}^{2}+\sigma_{E_{\mathrm{cr}}}^{2}}}\right)$$

#### 3.3.2. First-Order Second-Moment Method

The first-order second-moment method is the simplest method in reliability analysis [29]. This method linearizes the nonlinear performance function and then calculates the first and second moments of the linearized function through the first and second moments of basic variables, thereby obtaining the approximate failure probability of the performance function. Based on the first-order second-moment method, the relevant parameters involved in the service life prediction of MOCRC specimens are expressed as shown in Equations (17)–(19).

(17) $$\begin{array}{lll} \frac{\partial E_{r}}{\partial t} & = & 0.9226\times \left(2.2273-0.0147f\right)\times \left(1.8337-0.182\ln L\right)/\left(t+0.0074f+0.2843\ln L+0.332\right)\\ & + & 0.9426\times \left(0.0117f-0.9914\right)\times \left(2.2515-0.272\ln L\right) \end{array}$$

(18) $$\begin{array}{lll} \frac{\partial E_{r}}{\partial f} & = & 0.0068\times \left(2.2273-0.0147f\right)\times \left(1.8337-0.182\ln L\right)/\left(t+0.0074f+0.2843\ln L+0.332\right)\\ & - & 0.0136\times \left(1.8337-0.182\ln L\right)\times \ln\left(t+0.0074f+0.2843\ln L+0.332\right)\\ & + & 0.011\times \left(2.2515-0.272\ln L\right)\times t \end{array}$$

(19) $$\begin{array}{lll} \frac{\partial E_{r}}{\partial L} & = & -0.1679\times \left(2.2273-0.0147f_{t}\right)\times \ln\left(t+0.0074f_{t}+0.2843\ln L+0.332\right)/L\\ & + & 0.2623\times \left(2.2273-0.0147f_{t}\right)\times \left(1.8337-0.182\ln L\right)/\left(t+0.0074f_{t}+0.2843\ln L+0.332\right)/L\\ & - & 0.2564\times \left(0.0117f_{t}-0.9914\right)\times t/L \end{array}$$

#### 3.3.3. Service Life

(1) Service life of MOCRC

According to reliability theory and the first-order second-moment method, the service life of MOCRC specimens can be obtained based on the time-varying degradation of MOCRC. Studies [28] show that concrete fails when the RDEM of concrete is less than 0.60. Therefore, 0.60 serves as the critical value for MOCRC failure. The related parameters of deterioration behavior for concrete follows a normal distribution [24], and the variation coefficient is 5%. The normal distribution parameters for predicting the service life of MOCRC specimens are listed in Table 6.

**Figure 9:**
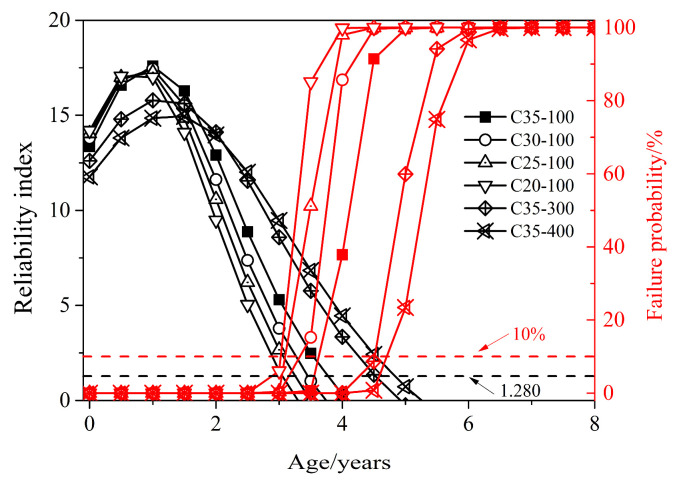
Relationship between the failure evaluation parameters and service ages of MOCRC.

The relationship between the failure evaluation parameters and the service ages of MOCRC specimens in a western saline soil environment is shown in Figure 9. It can be seen from Figure 9 that as the service age increases, the reliability of MOCRC specimens first increases and then decreases. The reason for the decrease in the reliability is the transformation of the 518 phase and MgO into Mg(OH)_2_ in MOCRC specimens, which is confirmed by the results in Figure 1 and Figure 2. Moreover, the failure probability of MOCRC specimens gradually increases with the increase in service age. When the maximum failure probability is 10%, corresponding to a reliability index of 1.280, the service life of MOCRC specimens gradually shifts to the left as the increase in the compressive strength or length decreases.

Table 7 shows the service life of MOCRC specimens in a western saline soil environment with different compressive strengths and lengths. When the length of MOCRC specimens is the same, the service life of C30-100, C25-100, and C20-100 reduces by 8.3%, 14.9%, and 16.8% compared with C35-100, respectively. When the compressive strength of MOCRC specimens is the same, the service lives of C35-300 and C35-400 increase by 24.2% and 29.5% compared with C35-100, respectively. In summary, the service life of MOCRC specimens in a western saline soil environment gradually increases with the increase in compressive strength or length, which is consistent with the deterioration of MOCRC. It should be noted that the above-mentioned MOCRC specimens cannot meet the minimum service life requirement of 50 years for concrete structures in harsh environments.

(2) Service life of extrapolated MOCRC (E-MOCRC)

The above results show that an increase in the compressive strength or length can increase the service life of MOCRC. To meet the practical engineering requirements, the strength grade of MOCRC should be C60, which is recorded as E-MOCRC in this paper. Assuming that the length of E-MOCRC is 400 mm, 1000 mm, 2000 mm, and 3000 mm, the normal distribution parameters for predicting the service life of E-MOCRC specimens are listed in Table 8.

**Figure 10:**
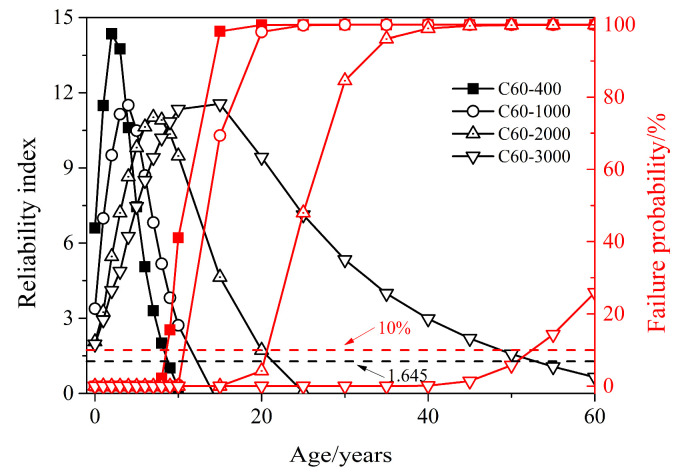
Relationship between the failure evaluation parameters and service ages of E-MOCRC.

Figure 10 shows the trend of failure probability and reliability of E-MOCRC with the increase in service age, which is consistent with that of MOCRC. As the service age increases, the reliability of E-MOCRC first increases and then decreases. Moreover, the failure probability of E-MOCRC specimens gradually increases with the increase in service age. When the maximum failure probability is 10%, corresponding to a reliability index of 1.280, the service life of E-MOCRC specimens gradually shifts to the left as the increase in compressive strength or length decreases.

From Table 9, the service life of C60-1000, C60-2000, and C60-3000 increases by 24.7%, 140.8% and 511.5% compared with C60-400, respectively. When the size is the same, the service life of C60-400 increases by 82.6% compared with C35-400. The results show that both compressive strength and length have a significant effect on the service life of E-MOCRC. When the compressive strength is 60 MPa and the length is 3000 mm, MOCRC can meet the minimum requirement of a service life of 50 years for concrete structures in harsh environments.

## 4. Conclusions

(1) In a simulated western saline soil environment, the RDEM of MOCRC specimens increases and then decreases with the increase in service age. In addition, as the compressive strength or length decreases, the RDEM of MOCRC specimens decreases, which indicates that the durability of MOCRC decreases.

(2) There is a clear functional relationship between the relative dynamic elastic modulus and compressive strength or size of MOCRC. Based on this relationship, a multiparameter time-varying degradation model for MOCRC is proposed with consideration of compressive strength, size effect, and environmental error. According to reliability theory and the first-order second-moment method, a service life prediction model for MOCRC is established based on the relative dynamic elastic modulus.

(3) In this paper, C60-3000 can meet the minimum requirement of a service life of 50 years for concrete structures in western saline soil environments, but the others cannot meet this requirement. Therefore, the durability of MOCRC structures in western saline soil environments still needs further research. It should be emphasized that the proposed model indicates trends rather than validated design values; further field validation is required.

## Figures and Tables

**Table 1:** Chemical composition of LBM and FA.

Material	MgO/%	SiO_2_/%	CaO/%	Fe_2_O_3_/%	SO_3_/%	Na_2_O/%	K_2_O/%	Al_2_O_3_/%	Loss/%	Others/%
LBM	80.64	0.51	3.24	0.62	0.21	0.42	0.49	-	0.98	12.89
FA	4.26	57.29	2.40	4.39	0.39	-	-	25.86	0.96	4.45

**Table 2:** Performance indicators of fine and coarse aggregates.

Material	Apparent Density /kg·m^−3^	Loose Bulk Density /kg·m^−3^	Clay Content /%	Porosity /%	Moisture Content /%
Sand	2610	1600	2.4	38.9	2.7
CA	2780	1520	0.5	45.3	0.3
RCA	2490	1410	0.7	51.2	3.0

**Table 3:** The mix ratios and 28-day compressive strength of MOCRC/kg·m^−3^.

No.	LBM	MC	FA	Sand	CA	RCA	PA	NS	Water	28-Day Compressive Strength/MPa
C35	389	148	68.5	625	1170	0	4.5	16	146	36.0
C30	389	148	68.5	625	877.5	292.5	4.5	16	146	31.2
C25	389	148	68.5	625	585	585	4.5	16	146	27.4
C20	389	148	68.5	625	292.5	877.5	4.5	16	146	23.4

**Table 4:** Chemical composition of western saline soil environment/g·L^−1^.

Na^+^	K^+^	Mg^2+^	Ca^2+^	Cl^−^	SO_4_^2−^	HCO_3_^−^
35.51	7.04	55.05	0.88	220.52	4.2	0.18

**Table 5:** Parameters of time-varying degradation fitting curves for MOCRC specimens.

No.	*a*	*b*	*c*	Adj. R^2^
C35-100	1.6844	1.7993	−0.5660	0.9786
C30-100	1.7863	1.7460	−0.6232	0.9664
C25-100	1.8377	1.7281	−0.6814	0.9779
C20-100	1.8688	1.7036	−0.7092	0.9847
C35-300	1.5491	1.8840	−0.4924	0.9360
C35-400	1.2434	2.2020	−0.3435	0.8921

**Table 6:** Normal distribution parameters for predicting the service life of MOCRC specimens.

No.	*f*/MPa	*L*/mm	*E*_cr_
C35-100	N(36.0, 1.80)	N(100, 5)	N(0.60, 0.03)
C30-100	N(31.2, 1.56)	N(100, 5)	N(0.60, 0.03)
C25-100	N(27.4, 1.37)	N(100, 5)	N(0.60, 0.03)
C20-100	N(23.4, 1.17)	N(100, 5)	N(0.60, 0.03)
C35-300	N(36.0, 1.80)	N(300, 15)	N(0.60, 0.03)
C35-400	N(36.0, 1.80)	N(400, 20)	N(0.60, 0.03)

**Table 7:** Service life of MOCRC.

No.	C35-100	C30-100	C25-100	C20-100	C35-300	C35-400
Service life/years	3.63	3.33	3.09	3.02	4.51	4.70

**Table 8:** Normal distribution parameters for predicting the service life of E-MOCRC.

No.	*f*/MPa	*L*/mm	*E*_cr_
C60-400	N(60.0, 3.0)	N(400, 20)	N(0.60, 0.03)
C60-1000	N(60.0, 3.0)	N(1000, 50)	N(0.60, 0.03)
C60-2000	N(60.0, 3.0)	N(2000, 100)	N(0.60, 0.03)
C60-3000	N(60.0, 3.0)	N(3000, 150)	N(0.60, 0.03)

**Table 9:** Service life of E-MOCRC.

No.	C60-400	C60-1000	C60-2000	C60-3000
Service life/years	8.58	10.70	20.66	52.47

## Data Availability

The original contributions presented in this study are included in the article. Further inquiries can be directed to the corresponding authors.
